# Mapping geographical areas at risk for tick-borne encephalitis (TBE) by analysing bulk tank milk from Swedish dairy cattle herds for the presence of TBE virus–specific antibodies

**DOI:** 10.1186/s13028-021-00580-4

**Published:** 2021-04-07

**Authors:** Gunilla Blomqvist, Katarina Näslund, Linda Svensson, Cécile Beck, Jean Francois Valarcher

**Affiliations:** 1 Department of Microbiology, National Veterinary Institute, 75189 Uppsala, Sweden; 2 Department of Disease Control and Epidemiology, National Veterinary Institute, 75189 Uppsala, Sweden; 3 UMR 1161 Virology, ANSES, INRAE, ENVA, ANSES Animal Health Laboratory, EURL for Equine Diseases, 94704 Maisons-Alfort, France; 4 Department of Clinical Sciences, Swedish University of Agricultural Sciences, Uppsala, Sweden

**Keywords:** Antibody detection, Bulk tank milk, Sentinel dairy cattle herds, Tick-borne encephalitis virus

## Abstract

**Background:**

The vector-borne human viral zoonosis tick-borne encephalitis (TBE) is of growing concern in Sweden. The area where TBE is considered endemic has expanded, with an increasing geographical distribution of *Ixodes ricinus* as the tick vector and a rising number of reported TBE cases in humans. Efforts to map TBE risk areas have been carried out by sentinel monitoring, mainly based on individual sampling and analysis of wild and domestic animals, as well as ticks, for tick-borne encephalitis virus (TBEV). However, the interpretation of the geographical distribution has been hampered by the patchy and focal nature of TBEV occurrence. This study presents TBEV surveillance data based on antibody analysis of bulk tank milk collected from dairy herds located throughout Sweden before (May) and after (November) the vector season. A commercial TBEV antibody ELISA was modified and evaluated for use in this study.

**Results:**

The initial comparative TBEV antibody analysis revealed a good correlation between milk and serum antibody levels from individually sampled cows. Also, the TBEV-antibody levels for the mean-herd serum showed good comparability with TBEV antibody levels from bulk tank milk, thus indicating good predictability of seroprevalence when analysing bulk tank milk from a herd.

Analyses of bulk tank milk samples collected from 616 herds in May and 560 herds in November showed a geographical distribution of TBEV seropositive herds that was largely consistent with reported human TBE cases. A few TBEV-reactive herds were also found outside known locations of human TBE cases.

**Conclusion:**

Serological examination of bulk tank milk from dairy cattle herds may be a useful sentinel surveillance method to identify geographical presence of TBEV. In contrast to individual sampling this method allows a large number of animals to be monitored. TBEV seropositive herds were mainly found in coastal areas of southern Sweden similar to human TBE cases. However, some antibody-reactive herds were found outside known TBE areas at the time of the study.

## Background

Tick-borne encephalitis (TBE) is considered the most important viral tick-borne human zoonosis in Eurasia [1], and since 2012 TBE has been included on the list of notifiable human diseases in the European Union [2]. In humans, TBE is often characterized by a biphasic course, with non-specific symptoms in the first phase and symptoms of the central nervous system in the second phase.

The causative agent of TBE is the tick-borne encephalitis virus (TBEV), a member of the genus Flavivirus within the family Flaviviridae [3]. TBEV, like louping ill virus, Powassan virus, and deer tick virus, belongs to the group of tick-borne flaviviruses known to cause central nervous system disorders in humans. Although TBEV-antibodies are prevalent in both wild and domestic animals, relatively few reports of TBE in animals are available. Clinical cases have been described in dogs [4–7], in one horse [8], and in one monkey [9]. Single cases of TBE have also been reported to occur in a sheep [10], a goat [11] and a mouflon [12].

There are three recognized subtypes of TBEV: the European virus (TBEV-Eu), the Far Eastern virus (TBEV-Fe), and the Siberian virus (TBEV-Sib) [13, 14]. However, whole-genome sequencing has recently provided evidence for the existence of six different TBEV subtypes [15].

The main vector for TBEV-Eu is the hard-bodied tick *Ixodes ricinus*, while TBEV-Fe and TBEV-Sib are mainly transmitted by *Ixodes persulcatus* [13]. The European subtype is thus far the only subtype found in ticks in Sweden, Denmark, and Norway. In Finland, however both the Siberian and the European subtypes have been found (both subtypes in the tick *I. persulcatus*), while only the European subtype is found in the tick *I. ricinus* [16].

The life cycle of *I. ricinus* includes three parasitic stages–larva, nymph, and adult–and each stage lasts for 1–2 years and sometimes up to 3 years [17].

In each developmental stage, the tick ingests blood only once, for a period of a few days, on hosts of different species [18, 19]. The adult ticks feed mainly on larger animals, such as roe deer, cattle, or sheep [19, 20]. Larvae feed mainly on small mammals (rodents) and birds. Nymphs also feed on small mammals, but like the adult tick, they also feed on larger host animals such as roe deer and hares.

TBEV is transmitted from viraemic host mammals to susceptible ticks (viraemic mode of transmission), or by the transmission of virions from infected ticks via phagocytic migratory blood cells to an uninfected tick feeding nearby on the same host animal (co-feeding) [21, 22]. The latter form of transmission is most often seen in rodents (non-viraemic mode of transmission) and usually involves transmission from infected nymphs to larvae [21, 23]. Generally, the virus is transferred from one tick stage to the next stage, and thus the tick remains infected throughout its whole life [13, 18, 24]. Transovarial transmission and transmission by mating ticks have also been described [25].

TBEV-Eu is mainly transmitted to humans and animals through tick-bites, primarily by *I. ricinus* nymphs [18, 19, 26]. Infection can also occur through consumption of unpasteurized milk from infected animals such as goats, sheep, and cows in the viraemic phase, during which time the virus is secreted with the milk [27–31].

TBE is of growing concern in Europe. A changing climate, with milder winters and earlier springs, has resulted in more favourable conditions for ticks and their hosts, and increased their presence and distribution range [18, 32, 33], thus increasing the potential for the spread of TBEV.

In Sweden, diagnosed human cases of TBE are reported to the county medical officers in accordance with the Communicable Diseases Act and then further reported to and registered by the Public Health Agency of Sweden. Since 1980s the number of human TBE cases has gradually increased and in 2017 the reported number of TBE cases was 391, the highest number of registered cases up to and including year 2020. In 2020 the number of registered TBE cases was 278 [18, 34]. The endemic area has expanded to the north and west as a result of the increasing abundance and expanded geographic range of *I. ricinus*, currently considered to be the only vector species for TBEV in Sweden [18]. This expansion of *I. ricinus* in Sweden and Europe is mainly attributed to the increasing number and range of roe deer, considered to be *I. ricinus* most important host [18, 33, 35]. Roe deer numbers temporarily declined in Sweden during the 2009–2010 winter due to severe cold but have increased rapidly since then. However, it is argued that small mammals (rodents) to a large extent serve as reservoir and amplifier hosts, while larger animals act as transportation and mating sites for the ticks [16, 18, 35, 36].

Attempts to map areas where TBEV is endemic through the use of sentinels have been made using serological studies of blood or milk from several different vertebrate animals [20, 36]. Also, the presence of virus/virus antigens has been analysed in small mammals, birds, collected ticks, and raw milk at risk of TBE [27, 36]. In these studies, the test material has been sampled from individual animals. Here we describe a possible alternative and, as far as we know, new approach that uses bulk tank milk samples from dairy farms as a mean of serological mapping of TBE risk areas.

During analysing it was found that the sensitivity of the commercially available enzyme-linked immunosorbent assay (ELISA) for detecting of TBEV-specific antibodies could be optimized by replacing the protein G conjugate with a developed and evaluated monoclonal antibody against bovine IgG_1_ (Boehringer Ingelheim Svanova, Sweden)_._ Since IgG_1_ represents the major Ig class in milk throughout the lactation period this conjugate was thought to be particularly useful in the detection of milk antibodies [37].

## Methods

### Bulk tank milk samples

Bulk tank milk surveys were conducted in Sweden before and after the 2012 vector season (April and November) to detect and monitor infection with Schmallenberg virus (SBV) [38]. Follow-up surveillance of bulk tank milk samples was conducted in May through June and again in November 2013. The sampled dairy farms were randomly selected from a list of Swedish milk producers which in 2013 included 4,867 dairy farms. A total of 616 tank milk samples from May 2013 and 560 tank milk samples from November 2013 were used for the TBEV analysis. A total of 554 tank milk samples from the selected farms were sampled both in May and in November 2013. The bulk tank milk samples were collected in vials containing bronopol as a preservative. On arrival at the National Veterinary Institute, the samples were centrifuged at 3000 RPM, after which the cream fraction was removed. After analysing for antibodies against SBV (see above), the samples were kept at − 18 °C until they were used for the TBEV antibody analysis.

### Individual sampling

**Fig. 1:**
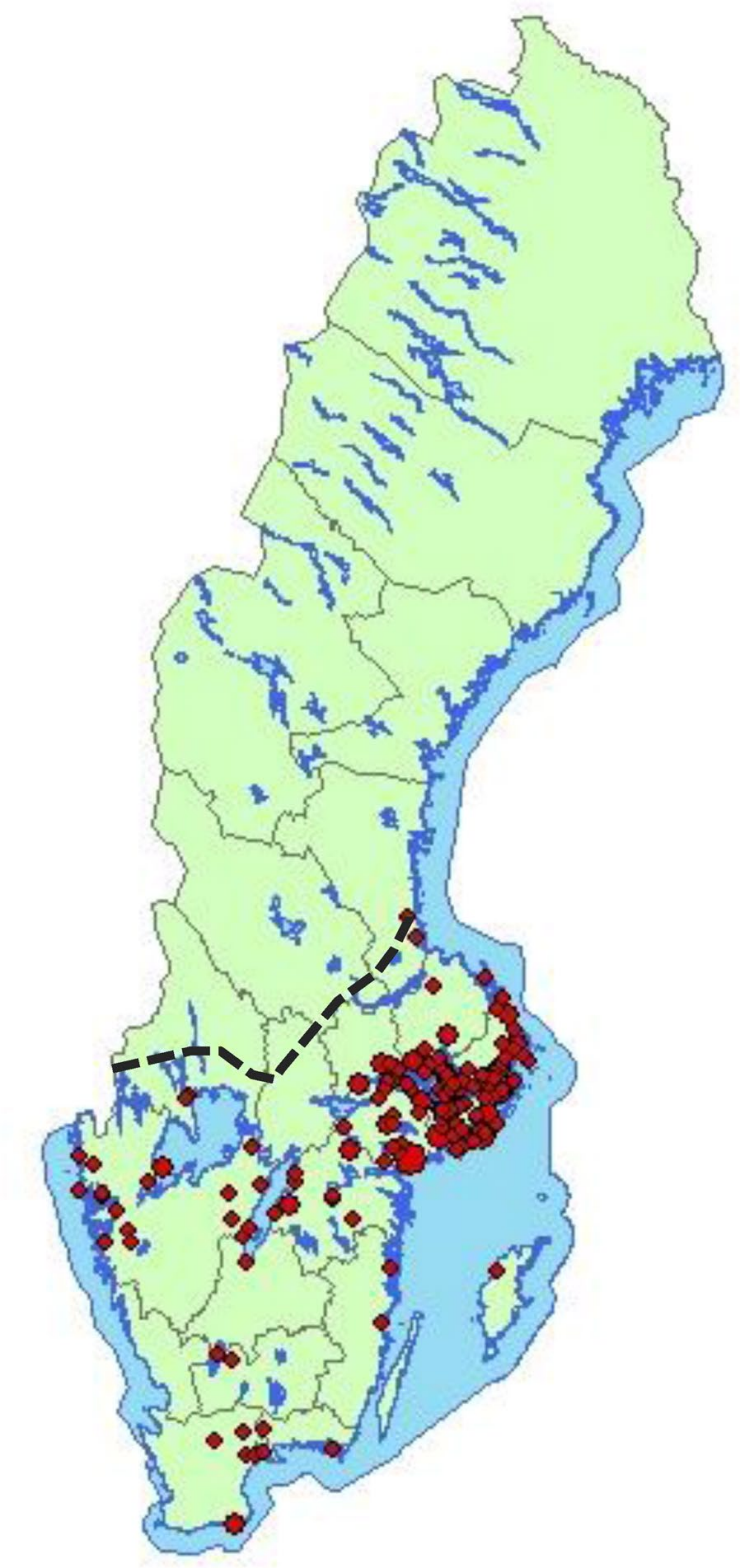
Geographical distribution of human TBE cases in 2013 Each dot represents one case of confirmed human TBE. The dashed line indicates the approximate borderline between Norrland and the rest of Sweden, known as the Limes Norrlandicus. (Map image courtesy of Marika Hjertqvist of the Swedish Public Health Agency)

Within the follow-up of SBV surveillance study in May 2013, individual cow blood samples were collected from 359 animals from 11 randomly selected dairy herds. In addition, individual milk samples from 108 cows were collected from 4 out of the 11 herds. Except for two herds in the counties of Värmland and Jämtland, all individually sampled herds were located south of the border zone between Norrland and the rest of Sweden (known as the Limes Norrlandicus); the region of Sweden where the vast majority of human cases of TBE have been reported (Fig. 1).

### Microneutralization test

Serum samples were analysed for virus-neutralizing antibodies in a virus-specific microneutralization test (MNT). Heat-inactivated serum was serially diluted (1/5 to 1/320) and mixed with an equal volume (50 µL) of DMEM containing 100 tissue culture infectious doses 50 (TCID_50_) of TBEV hypr strain (genbank code: U39292.1). After incubating the plates at 37 °C for 1.5 h, 2 × 10^4^ Vero cells in 100 µL of DMEM were added to every well. The plates were incubated at 37 °C for 5 days, then cytopathogenic effects were observed under a light microscope. The results were considered validated if (i) infected cells were absent in the cell controls, (ii) infected cells were present in the virus controls, (iii) the virus titre was between 75 and 125 TCID_50_ per well, (iv) no protective effect was seen with the negative reference serum, and (v) the positive reference serum protected the Vero cells from infection. A serum was considered negative if cells were found infected at any serum concentration. A serum was considered positive if cells were protected at least at the 1/20 serum dilution; its titre was calculated as the inverse of the last dilution at which the cells were protected.

### Commercial indirect ELISA (cE) for the detection of TBE antibodies

A commercial indirect ELISA (cE) (Immunozym FSME (TBE) IgG All Species, Progen, Germany), based on inactivated TBEV and a Protein G conjugate, was initially used for analysis. The tests were performed, and the results interpreted in accordance with the manufacturer’s protocol.

### Modified indirect ELISA (mE) for detection of TBE antibodies

Due to low conformity between the TBEV cE and MNT, a modified protocol for indirect ELISA was implemented. The protein G conjugate included in the commercial indirect ELISA kit was replaced with an HRP conjugated monoclonal antibody anti-bovine IgG_1_ (Boerhringer Ingelheim Svanova, Sweden). Instead of the human control sera included in the kit, bovine sera were used as controls. A positive control was made up of a pool of 8 positive bovine sera with a concentration of 260 VIEU (Vienna units)/mL in Progen TBE ELISA (> 126 VIEU/mL is considered positive). The negative control consisted of a pool of 22 negative bovine sera analysed in Progen TBE ELISA. The low positive control was made up of the positive control diluted 1:4 in the negative control.

The assay procedure for the Progen ELISA kit protocol was then followed, with minor modifications of the conjugate and substrate incubation steps. Bovine control and test sera were diluted 1:50 while the defatted milk/bulk milk samples were tested without dilution. Samples and a blank (buffer) were added in duplicate, in volumes of 200 µL, into the wells of the TBE coated Progen ELISA. They were then incubated for one hour at room temperature. The wells were then washed three times, and 200 µL of the anti-bovine IgG_1_ conjugate diluted 1:20,000 was added. The 1:20,000 dilution was used based on a previously developed ELISA for detection of SBV antibodies in ruminants, where the optimal dilution of the IgG_1_ conjugate was estimated [37]. After incubation for one hour at + 37 °C followed by washing three times, the substrate was added (200 µL) and incubated for 10 min. The reaction was stopped, and the optical density was measured at 450 nm. The mean OD value for each sample minus the OD value of the blank was calculated, and the result was expressed as ratio of the sample and positive control sera mean OD values multiplied by 100 (S/P %). The prerequisites for a valid result were an OD value for the positive control of ≥ 1.2, a S/P% value for the low positive control of 20–30, and a S/P% value for the negative control of < 10.

### Estimation of cut-off values for modified indirect ELISA (mE)

Estimation of cut-off values was based on analysing presumed TBEV seronegative milk and serum sampled from 23 cows from a dairy herd located within a hitherto TBE-free area of northern Sweden (county of Jämtland). The cut-off values of negative serum and milk samples were calculated by adding the mean S/P % value and two standard deviations from the mean S/P % value of the serum and milk samples, respectively.

### Correlation between individual serum and milk TBEV antibody levels

Individual serum and milk samples from 108 cows from 4 herds were analysed with TBEV mE.

The relationship between serum and milk TBEV antibody levels was then evaluated using Pearson correlation test.

### Correlation between mean-herd serum and bulk tank milk TBEV antibody levels for the corresponding herds

Individual serum and bulk tank milk samples from 359 animals from 11 herds were analysed with TBEV mE. The relationship between mean-herd serum and bulk tank milk TBEV antibody levels was then evaluated using Pearson correlation test.

## Results

### Estimation of cut-off values for TBEV modified indirect ELISA (mE)

The analysis data used to calculate the cut-off values were based on 23 serum and milk samples from cows living in a county hitherto known as a TBEV-free area. The analyses gave a mean S/P % value of 4.7 and a standard deviation (SD) value of S/P % 3.9 in serum and a mean S/P % value of 4.9 and a SD value of S/P % 3.7 in milk. Thus, the cut-off point of the TBEV mE was estimated to be S/P % 12 in both serum and milk samples. Analysis results with a S/P % value below 12 were therefore defined as negative.

### Comparative analysis of serum TBEV-antibody levels using microneutralization test (MNT), commercial indirect ELISA (cE) and modified indirect ELISA (mE)

Nine bovine sera with varying MNT titres were analysed with TBEV cE and TBEV mE. Out of the 8 MNT-positive sera, the TBEV cE analysis results of 2 were positive, 2 were doubtful, and 4 were negative (Table 1).

**Table 1:** Comparison of the results of micro neutralization test (MNT) commercial indirect ELISA (cE) and modified indirect ELISA(mE) for the detection of TBEV antibodies in bovine serum

Sera	MNT	cE	mE
No	titre	VIEU/mL	S/P %
1	*1:80*	*139*	*179*
2	*1:40*	29	*25*
3	*1:320*	*111*	*114*
4	*1:20*	30	11
5	*1:40*	22	*23*
6	*1:160*	*91*	*118*
7	*1:80*	33	*50*
8	*1:40*	*161*	*21*
9	< 1:20	25	*19*

Italic indicates positive results, italic underline indicates doubtful results

MNT titre ≥ 1:20 = positive

cE > 126 = positive, 63–126 doubtful and < 63 negative

mE > 19 = positive, 12–19 doubtful, < 12 negative

**Fig. 2:**
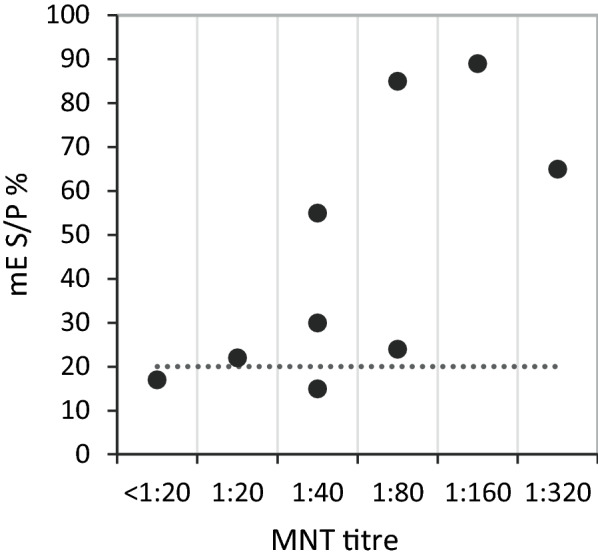
Comparative analysis of serum for TBEV antibodies with microneutralization test (MNT) and modified indirect ELISA (mE). The dotted line indicates an S/P % value of 20 in mE. Of the MNT positive sera, 7 of 8 got S/P % values of ≥ 20 with mE. One positive serum with an MNT titre of 1:40 got an S/P % value of 15, while one negative MNT serum got an S/P% value of 17

The comparison between MNT and S/P % values for the TBEV mE analyses is presented in Table 1 and Fig. 2.

The results showed that serum samples with a S/P % value of ≥ 20 correlated with MNT positive serum samples except for one sample which was positive in MNT but negative in TBEV mE.

By setting S/P % values of ≥ 20 as positive and S/P % values of < 12 as negative, S/P % values between 12 and 19 were considered as doubtful when analysing serum samples for TBEV-antibodies with the TBEV mE.

### Correlation between individual serum and milk TBEV antibody levels

**Fig. 3:**
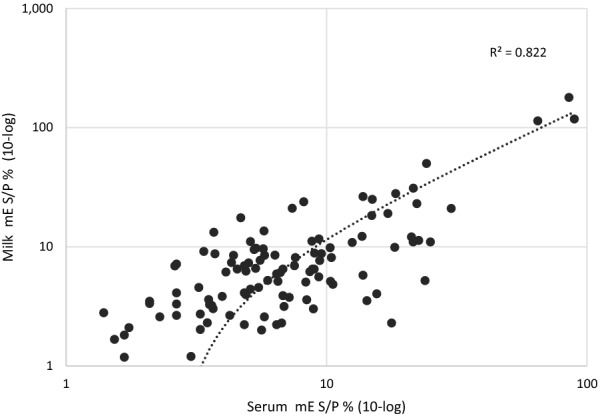
Correlation between individual serum and milk TBEV antibodies. The dot plot graph illustrates the modified indirect ELISA (mE) S/P % values of the analysed milk and serum individually sampled from 108 cows from 4 herds. Pearson correlation test yields a coefficient of determination of R^2^ = 0.822; (P < 0.001)

The relationship between TBEV antibody levels of serum and milk from 108 individually sampled cows from 4 herds was evaluated using Pearson correlation test. The results indicated a significant correlation between individual serum and milk TBEV antibody levels (r (106) = 0.91, P < 0.001). The coefficient of determination was R^2^ = 0.822; (P < 0.001), see Fig. 3.

### Correlation between mean-herd serum TBEV antibody levels and TBEV antibody levels in bulk tank milk for the corresponding herds

**Fig. 4:**
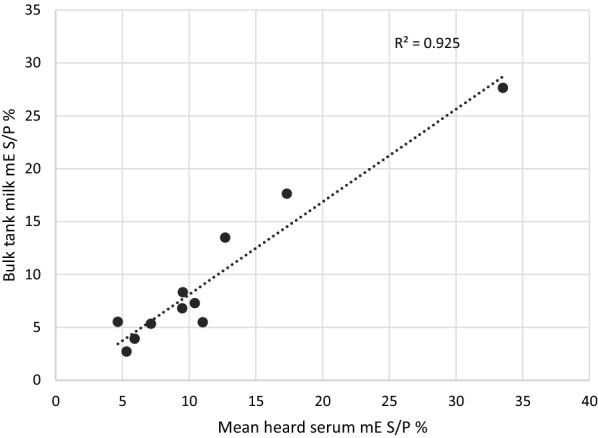
Correlation between mean herd serum and bulk tank milk TBEV antibodies. The dot plot graph illustrates the modified indirect ELISA (mE) S/P % values of the analysed bulk tank milk and the mean herd serum sampled from 359 cows from 11 herds. Pearson correlation test yields a coefficient of determination of R^2^ = 0.925; (P < 0.001)

Serum and bulk tank milk samples from eleven herds, representing 359 animals altogether, were analysed with TBEV mE. The bulk tank milk S/P % value was < 12 (between 3 and 8) for 8 of the 11 herds, while 2 herds yielded a S/P % value of 13 and 18, respectively. Bulk tank milk from one herd yielded a S/P % value of 28. The average serum antibody level for each herd was calculated by summing the TBEV mE S/P % results and dividing the sum by the number of serum samples analysed. Results of the Pearson correlation test indicated a significant correlation between the TBEV antibody levels of the mean-herd serum and the bulk tank milk (r (9) = 0.96, P < 0.001). The coefficient of determination was R^2^ = 0.925; (P < 0.001), see Fig. 4.

### TBEV antibody analysis of bulk tank milk samples

**Fig. 5:**
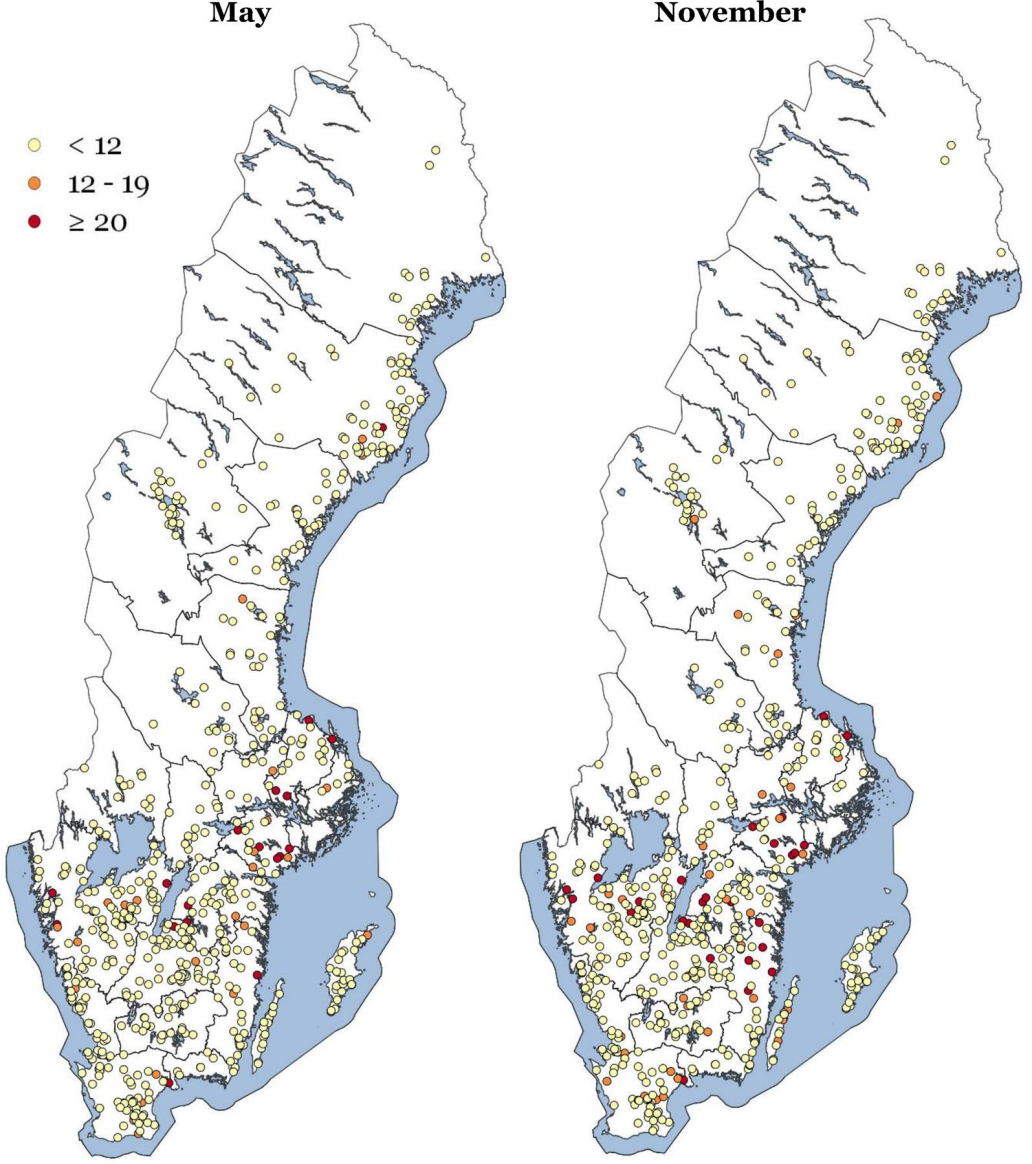
Geographical distribution of Swedish dairy herds from which bulk tank milk was sampled in May and November 2013 for TBEV antibody analysis using modified indirect ELISA (mE). Each dot represents one dairy herd. The results from the mE analysis are divided into three groups represented by three different coloured dots. mE S/P % values of < 12 are considered negative, 12–19 doubtful, and ≥ 20 positive

Figure 5 and Table 2 present the results of the bulk tank milk samples analysed using TBEV mE. As shown, the number of TBEV seropositive herds increases between May and November, which can be expected based on the increase in tick feeding activity as temperatures rise across the course of the summer. Table 3 shows the TBEV antibody levels for 554 herds sampled both in May and in November.

**Table 2:** TBEV modified indirect ELISA (mE) S/P % value of all bulk tank milk samples collected in May and November

S/P % values	Number of bulk tank milk samples/herds	
	May	November
< 12	570	490
12–19	28	43
≥ 20	18	27
Total number of herds	616	560

Samples were analysed in modified indirect ELISA (mE)

S/P % values of < 12 are considered negative, S/P % values of 12–19 doubtful, and ≥ 20 positive, for the presence of antibodies

**Table 3:** TBEV modified indirect ELISA (mE) S/P % value of paired bulk tank milk samples collected in May and November

S/P % values	Number of bulk tank milk samples/herds	
	May	November
< 12	514	484
12–19	23	43
≥ 20	17	27
Total number of herds	554	554

Samples were analysed in modified indirect ELISA (mE)

S/P % values of < 12 were considered negative, S/P % values of 12–19 doubtful, and ≥ 20 positive, for the presence of antibodies

In 4 out of 17 herds with bulk tank milk antibody levels of S/P % ≥ 20 in May, bulk tank milk antibody levels were lower in November, while in 14 herds these same antibody levels increased from S/P % < 20 to S/P % ≥ 20. The number of doubtful herds increased from 23 to 43 out of 554 between May and November. In 13 herds, the tank milk antibody level was S/P % ≥ 20 both in May and in November.

## Discussion

The present study describes a serosurveillance study of Swedish dairy cattle herds for use in sentinel monitoring of TBEV. Bulk tank milk samples collected in May and November 2013 were analysed to detect antibodies against TBEV. The results for geographic distribution of TBEV seropositive dairy herds mainly aline with areas of reported human cases of TBE and like human cases were more prevalent in the coastal areas of southern Sweden (Figs. 1, 5). However, some antibody-reactive herds were found outside known TBE areas at the time of sampling, i.e., in the northern counties Västerbotten and Jämtland. After 2013 and until 2021 TBE was reported from the county of Jämtland in 2015 (one case), 2017 (two cases) and 2018 (one case), and from the county of Västerbotten in 2017 (two cases) and 2019 (one case) [34].

Comparative analysis showed a reasonable predictive correlation between antibody levels in bulk tank milk and mean serum antibody levels within a herd. However, antibody analyses of bulk tank milk from a herd should be evaluated with careful consideration according to the dilution effect resulting from the ratio of seropositive to seronegative animals, as well as the individual antibody levels in seropositive animals. Even in herds with bulk tank milk defined as seronegative (S/P % ≤ 20), we found individual positive samples (15 out of 269). Therefore, especially when doubtful results are obtained, further examination is merited through individual analyses or analyses of smaller pools of milk or serum samples in order to clarify the presence of TBEV seropositive animals.

Although the use of tank milk samples for sentinel monitoring has limitations in terms of sensitivity, it nevertheless offers significant advantages. Sampling is easy to implement: one sample represents several individuals who may have been exposed to infection. Sampling at the end of the grazing season or vector period maximizes the time of exposure to infection: TBEV antibody analysis of bulk tank milk samples from 554 dairy farms sampled both in the spring (May) and in the fall (November) showed a higher incidence of TBEV antibodies in the fall (4.8%) compared to spring (3.0%). In addition, mapping of TBEV presence can be integrated into existing national control programs by using available tank milk samples collected for serosurveillance of bovine diseases.

The irregular distribution and patchy geographical occurrence of TBEV [16, 39] may hamper sentinel monitoring at an individual level, something several surveys have also reported [36]. Analysis of bulk tank milk sampled from dairy farms may therefore offer an easier and more comprehensive approach for monitoring TBEV prevalence, and likely other tick-borne viruses, within a given geographical region.

## Conclusion

The results of this study indicate that serological examination of bulk tank milk from dairy cattle herds may be useful as a surveillance strategy to identify geographical presence of TBEV. It was found that TBEV seropositive dairy herds like human cases of reported TBE, were more prevalent in coastal areas of southern Sweden. Some antibody-reactive herds were found outside known TBE areas at the time of the study. In contrast to surveillance based on individual animals this method allows a large number of animals to be monitored thereby increasing the probability of finding the presence of TBEV. Also, by sampling at the end of the grazing season or vector period the time of exposure to infection is maximized. Sampling is easy to implement and may be integrated into national control programs for serosurveillance of bovine diseases.

## Abbreviations

cE: Commercial indirect ELISA,
DMEM: Dulbecco′s Modified Eagle′s–Medium
ELISA: Enzyme-linked immunosorbent assay
HRP: Horseradish peroxidase
*I. persulcatus*: *Ixodes persulcatus*
*I. ricinus*: *Ixodes ricinus*
MNT: Microneutralization test
mE: Modified indirect ELISA
SBV: Schmallenberg virus
S/P %: Unit for test result calculated as the ratio between Sample result and Positive control sera expressed as percentage. The unit expresses the relative amounts of antibodies in a sample
TBE: Tick-borne encephalitis
TBEV: Tick-borne encephalitis virus
TBEV-Eu: European tick-borne encephalitis virus
TBEV-Fe: Far Eastern European tick-borne encephalitis virus
TBEV-Sib: Siberian European tick-borne encephalitis virus
TCID50: Tissue culture infectious doses 50
VIEU: Vienna units
